# Guillain–Barré Syndrome (42 Cases) Occurring During a Zika Virus Outbreak in French Polynesia

**DOI:** 10.1097/MD.0000000000003257

**Published:** 2016-04-08

**Authors:** Louise Watrin, Frédéric Ghawché, Philippe Larre, Jean-Philippe Neau, Stéphane Mathis, Emmanuel Fournier

**Affiliations:** From the Department of Neurology (LW, J-PN, SM), Poitiers University Hospital Center, Poitiers, France; Department of Neurology (FG, PL), French Polynesia Hospital Center, Papeete, Tahiti, French Polynesia; and Department of Clinical Neurophysiology (EF), La Pitié-Salpêtrière University Hospital Center, AP-HP, Groupe Hospitalier Pitié-Salpêtrière, Bd de l’Hôpital, Paris cedex, France.

## Abstract

Zika virus (transmitted by mosquitoes) reached French Polynesia for the first time in 2013, leading to an epidemic affecting 10% of the total population. So far, it has not been known to induce any neurological complications, but, a few weeks after the outbreak, an unexpectedly high number of 42 patients presented with Guillain–Barré syndrome.

We report the clinical and electrophysiological characteristics of this series. Males predominated with a sex ratio of 2.82 (mean age: 46). All patients (except 2) were native Polynesian. At admission, 55% were able to walk unaided against 38% at nadir, 24% had swallowing troubles (nadir: 45%), 74% had motor weakness of the limbs (nadir: 86%) and deep tendon reflexes were diminished or not found in the vast majority of patients. Mean duration of the progressive phase and of the plateau phase was respectively 7 and 9 days. Thirty-eight percent of the patients were admitted in intensive care unit and 10 patients underwent tracheotomy. Nerve electrophysiological studies at admission showed marked distal motor conduction alterations, which had almost completely disappeared at the 4th month; this pattern was more suggestive of acute motor axonal neuropathy (AMAN) than of acute inflammatory demyelinating polyneuropathy (AIDP). Lumbar puncture showed elevated proteins in 90% of the cases, with cell count always inferior to 50/μL.

This epidemic raises several questions, such as the potential existence of interactions between Zika virus and Polynesian HLA system and/or the consequences of several recombination events of this virus. This situation should call for increased vigilance, especially in countries where *Aedes* mosquitoes are present.

## INTRODUCTION

Guillain–Barré syndrome (GBS) is an immune-mediated inflammatory neuropathy occurring at all ages and requiring early diagnosis for accurate monitoring and treatment. Its annual incidence ranges from 0.89 to 1.89 per 100,000 persons in Western countries.^1^ It is commonly characterized by a rapidly progressive bilateral sensorimotor polyneuropathy (progressing over several days to 4 weeks), but the clinical spectrum of GBS is heterogeneous with various subtypes.^1^ GBS is usually triggered by infections or vaccinations,^2^ suggesting an immune origin.^1^ The most frequently associated pathogen is *Campylobacter jejuni* (30%).^3^ Tropical pathogens such as flaviviruses are potentially able to affect the nervous system, but most of these neurological manifestations are encephalitic.^4^ Only a few sporadic cases of GBS have been reported secondary to dengue virus (DENV) infection,^5,6^ West Nile virus (WNV) infection,^7^ and Japanese encephalitis virus (JEV) outbreaks.^8^

ZIKV is a single-stranded RNA-enveloped arthropod-borne virus transmitted by mosquitoes. It belongs to the *Flaviviridae* family, phylogenetically related to DENV, WNV, or JEV viruses.^9^ This pathogen was first isolated in 1947 from a febrile sentinel rhesus monkey in the Zika forest in Uganda.^10^ The sylvatic cycle mainly involves mosquitoes (*Aedes*), monkeys, and occasionally humans.^11^ Cases of Zika fever have sporadically appeared in some African countries and in parts of Asia.^11,12^ In 2007, the epidemic capacity of ZIKV was revealed in the southwestern Pacific Ocean.^12^ However, the largest reported outbreak of ZIKV was experienced in 2013 to 2014 in the South Pacific, in French Polynesia (28,000 cases; 10% of the total population).^13^ The strain of ZIKV isolated in French Polynesia was closely related to Yap State 2007 and Cambodia 2010, supporting an expansion of lineage of ZIKV.^14^ ZIKV is not known to provoke neurological complications.^15^ To our knowledge, before the Polynesian outbreak, there was no case of GBS after Zika virus (ZIKV) infection was reported. We aim to report the clinical and electrophysiological characteristics of the first and largest series of Guillain–Barré syndrome (GBS) possibly related to ZIKV (42 cases).

## MATERIAL AND METHODS

### Setting

French Polynesia is a French overseas territory made up of 119 islands distributed into 5 archipelagos: Society (encompassing Tahiti island), Marquesas, Tuāmotu, Gambier, and Austral Islands. The overall population is about 270,000 inhabitants living on 67 islands spread out over an area close to the size of continental Europe. In French Polynesia, two-thirds of the population are ethnically unmixed Polynesians, the rest of the population being composed of mixed Polynesians, Europeans (Caucasians), or East Asians.^16^ In French Polynesia, surveillance for acute illnesses is coordinated by the Department of Health, the main public hospital (Centre Hospitalier Territorial du Taaone in Pirae; Tahiti island) and the Public Health and Research Institute (Institut Louis Malardé).^14^

### Patients

The study included all of the patients who fulfilled the recently validated diagnostic criteria for GBS^17^ developed by the Brighton Collaboration;^18^ all of them came from Tahiti and were admitted in the Department of Neurology in Tahiti's hospital from November 2013 (week 45) to April 2014 (over the course of the Zika outbreak that had begun in week 41, in October 2013) where local neurologists excluded other neurological diagnoses (such as meningitis, encephalitis, and myelitis) following routine diagnostic work-up (clinical examination, lumbar puncture, ancillary tests, electrophysiological study, and radiological imaging if necessary): clinical and electrophysiological data were collected retrospectively; biological and radiological (brain CT-scan, brain and spine MRI) data were also collected for differential diagnosis. Written informed consent was obtained from all patients.

### Demographical and Clinical Data

Age, sex, ethnicity, and comorbidities were noted. We asked all patients whether they had a recent history of diarrhea, or zika/chikungunya/dengue virus infection. To clinically differentiate zika from dengue and chikungunya symptoms, we used the criteria from Yap State Department of Health Services and Halstead's criteria^19,20^ (Table 1). The clinical data were collected at entry and at nadir (defined as the highest GBS disability score). We evaluated gait disturbance 3 to 4 months after onset of GBS.

**TABLE 1:**
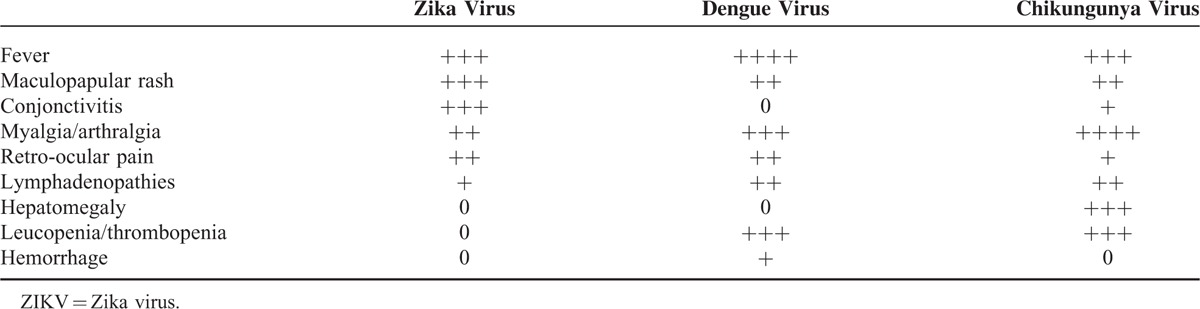
Main Clinical Characteristics of Zika Virus, Dengue Virus, and Chikungunya Virus Infections (Adapted From Yap State Department of Health Services and Halstead's Criteria)

### Electrophysiological Data

Electrophysiological studies of peripheral nerves were performed by the same practitioner on a multiple-channel apparatus (Micromed SystemPlus Evolution, Myoquick^®^), according to the standard electroneuromyography (ENMG) techniques.^21,22^

Compound muscle action potentials (CMAP) were recorded with surface electrodes from the median nerve (recording of the *abductor pollicis brevis*), the ulnar nerve (recording of the *abductor digiti minimi*), and the peroneal nerve (recording of the *extensor digitorum brevis*). Conventional methods were used for measurement of nerve conduction: latencies were measured at the onset of the action potential; amplitudes were measured from the baseline to the negative peak of the CMAP; distal CMAP durations and areas were measured from the onset of the initial negative phase to return to baseline of the last negative deflection of the CMAP.^23^ Topography of motor conduction abnormalities was classified as “distal” when distal latencies were > 110% of the upper limit of normal values (ULN) (or > 120% if distal amplitude was < 50% of the lower limit of normal values [LLN]); “intermediate” when conduction velocities were < 90% LLN (or > 50% if distal amplitude was > 50% LLN) or if there was a drop in the negative area of CMAP > 20% between distal and proximal stimulation sites (conduction block and abnormal dispersion were not distinguished).^24^ The terminal latency index (TLI) was also calculated to compare the distal segment with the intermediate segment for each nerve: TLI ≤ 0.25 was taken to indicate the presence of prominent distal motor slowing.^25^

Sensitive nerve action potentials (SNAP) were recorded for radial and sural nerves with surface electrodes. Sensory conduction velocities (SCV) were calculated from the onset latencies.

This ENMG protocol was performed twice on each patient: first during the first week after admission, and then during the 4th month. Two separate neurological medical teams, who worked blindly from each other, analyzed the data in Paris (France) and in Poitiers (France).

### Biological Data

A lumbar puncture was systematically performed for each patient. Acute phase serum and plasma samples were obtained during days 1 to 10 after the onset of the symptoms for serology and molecular assays. To exclude any ZIKV or DENV active infection, RT-PCR was carried out on blood samples and CSF;^26^ NS1 antigen was sought out in blood samples for DENV infection. As for ZIKV serology, at the time of the epidemic, techniques were still being developed and there was no kit available. Another relevant point is that cross-reactions with other flaviviruses are frequent and that French Polynesia was already experiencing a DENV (1 and 3) epidemic when the ZIKV epidemic started.^27^ As a result, ZIKV and DENV serology were not taken into account. Recent or active infections for the following common pathogens were also investigated: *C jejuni*, CMV, EBV, *Mycoplasma pneumonia*, *Chlamydia pneumonia,* HSV, HIV, hepatitis (A-B-C), leptospirosis, and syphilis.

Before treatment, plasma samples were sent to the Immunology laboratory of the Pitié-Salpêtrière’ Hospital (Paris, France), for detection of IgM and IgG to gangliosides (GM1, GM2, GD1a, GD1b, and GQ1b) with the ELISA technique (1:100 dilution and technique according to the manufacturer; Bühlmann-Gangliocombi^®^). They were considered negative when inferior to 30%, and positive when superior to 50% for each ganglioside.

### Statistical Analysis

Data were collected using EpiData software (EpiData Entry^®^; Denmark). Data could then be transposed to Excel tables (2003 version) in order to compile statistics. All continuous data were reported as means ± standard deviations (SD) or medians ± interquartile ranges (IQR: Q1-Q3). Categorical data were presented as proportions (%). Electrophysiological data were compared to normal values by *t* tests. Comparison of values obtained from the 2 successive ENMG assessments were performed by paired *t* tests. Comparison between GBS cases and ZIKV infections was performed with a Fisher's exact test.

## RESULTS

### Demographical Data

A total of 42 patients were included: males predominated (sex ratio = 2.82), and mean age was 46, ranging from 26 to 74 (median = 42). Out of the 42 patients, 39 came from the Society archipelago (29 from Tahiti, 6 from Moorea, 2 from Bora Bora, and 1 from Raiatea), 2 from the Tuamotu archipelago (Rangiroa and Makemo islands), and 1 from the Austral island of Tubuai. Among the 2 patients, 38 were native French Polynesian; 2 were born in New Caledonia, 1 in France, and 1 in French Guyana. All patients were in relatively good health.

### Clinical Data

**FIGURE 1:**
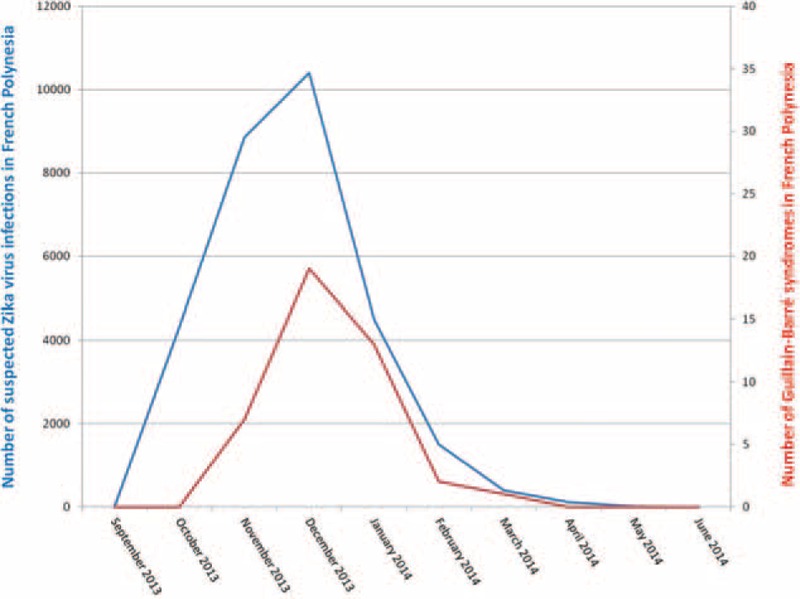
Epidemic curve of ZIKV infection suspected cases (in blue) and Guillain–Barré syndrome cases (in red) in French Polynesia (2013–2014); there is a statistical significative correlation between the number of patients in the 2 series: *p* < 0.05 (Fisher's exact test). ZIKV = Zika virus.

Thirty-seven patients (88%) recalled an infectious event within the month before the first neurological signs: 35 were more likely to have been ZIKV, 1 DENV, and 1 could have been either a DENV or ZIKV infection (no case evoked chikungunya). Among the cases of supposed ZIKV infection, symptoms were conjunctival hyperemia (40%), cutaneous eruption (80%), fever (49%), arthralgia (63%), and distal edema of the limbs (26%). The mean delay for initial neurological symptoms was 8 days (ranging from 2 to 23 days). The average delay between the onset of ZIKV epidemic and the onset of GBS outbreak was 4 weeks (Figure 1). Only 7 patients (17%) claimed to have had diarrhea within 2 months before admission.

The main clinical characteristics of GBS at baseline and at nadir are reported in Table 2. At admission, 55% of the patients were able to walk unaided. Sixteen patients (38%) were admitted in the intensive care unit. Of these, 10 underwent tracheotomy from 3 to 16 days after their admission (average = 7 days). Mean duration of stay was 39 days (3 to 127). During hospitalization, 15 patients (36%) had autonomic dysfunction with 1 having uncontrollable blood pressure.

**TABLE 2:**
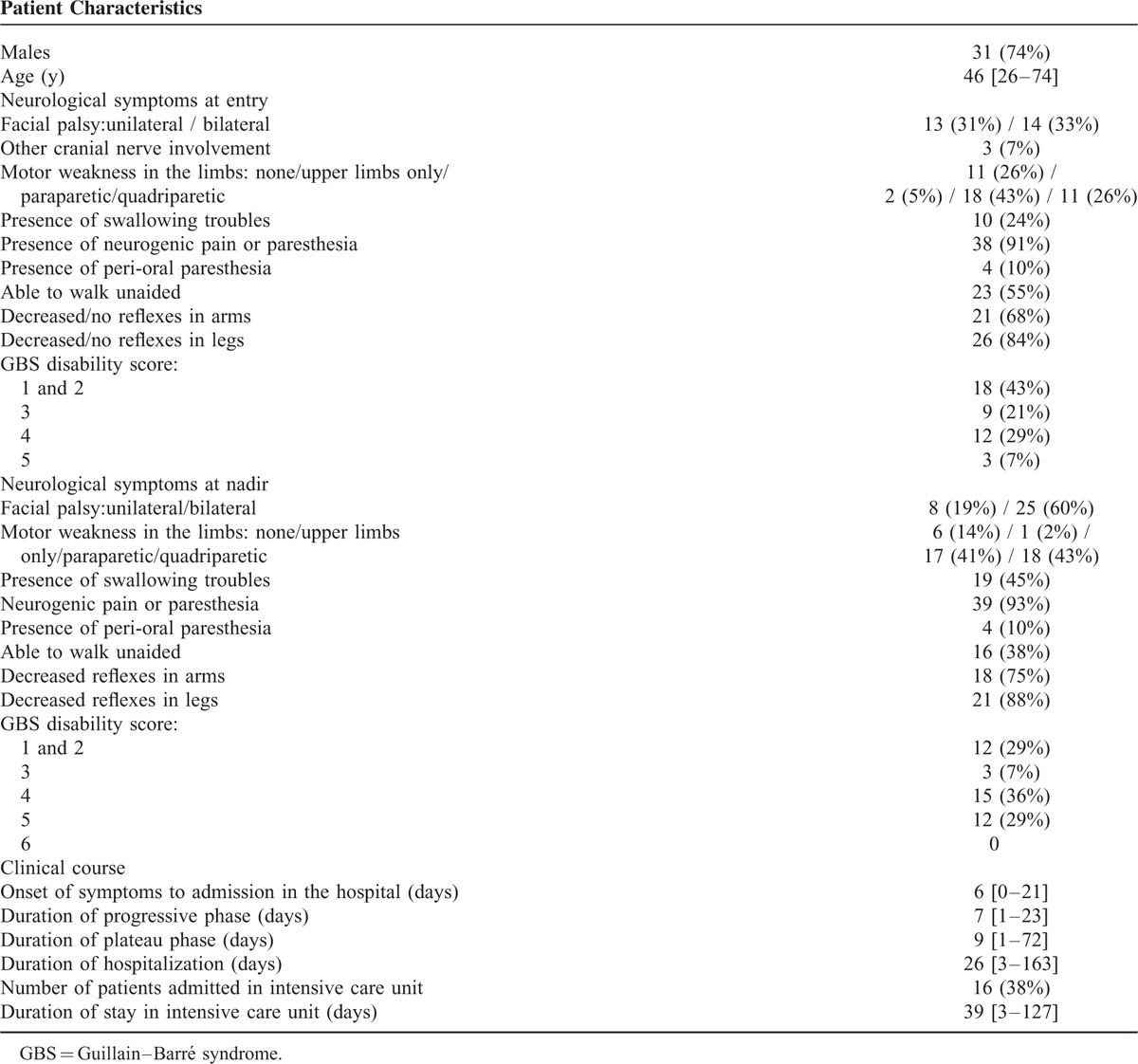
Clinical Description of Patients With Guillain–Barré Syndrome (n = 42)

All patients received IVIg at a dose of 2 g/kg of body weight over 2 to 5 days. Mean time between onset of symptoms and treatment was 6 days. Four patients received a second course of IVIg (same dose) because they were not clinically improving after the first. Plasma exchange was used in only 1 patient in the intensive care unit (4 sessions).

Mean duration was 7 days (ranging from 1 to 23 days) for the progressive phase, and 9 days (1 to 72 days; median of 4; IQR: 3–9.5) for the plateau phase. Average duration of hospitalization was 26 days (3 to 163 days; median of 12; IQR: 8–20). At discharge, 38% patients went home, and 60% to a rehabilitation center. Fifteen patients had a consultation 3 to 4 months after the beginning of their neurological signs: at that time, 8 patients (53%) were able to walk unaided.

### Electrophysiological Data

The main electrophysiological data are presented in Table 2. Thirty-seven of the 42 patients underwent ENMG assessment at admission. The average delay for this first electrophysiological study was 8 days after the onset of symptoms. Two had an “unexcitable” pattern (no stimulable sensory or motor nerves). In the 35 other cases, motor nerve conduction study showed almost the same pattern in all tested nerves, with prolonged distal latencies (*p* < 0.0001) and marked reduction of the distal compound muscle action potential (CMAP) amplitude (*p* < 0.0001), which is indicative of severe conduction alteration in the distal nerve segments. By contrast, there was no significant conduction slowing or block in the intermediate motor nerve segments (throughout forearm and legs). In all tested nerves, conduction abnormalities showed a primarily distal pattern, which was confirmed by TLI calculations (Table 3). Amplitude and conduction velocity of sensitive potentials were not significantly altered in the radial and sural nerves.

**TABLE 3:**
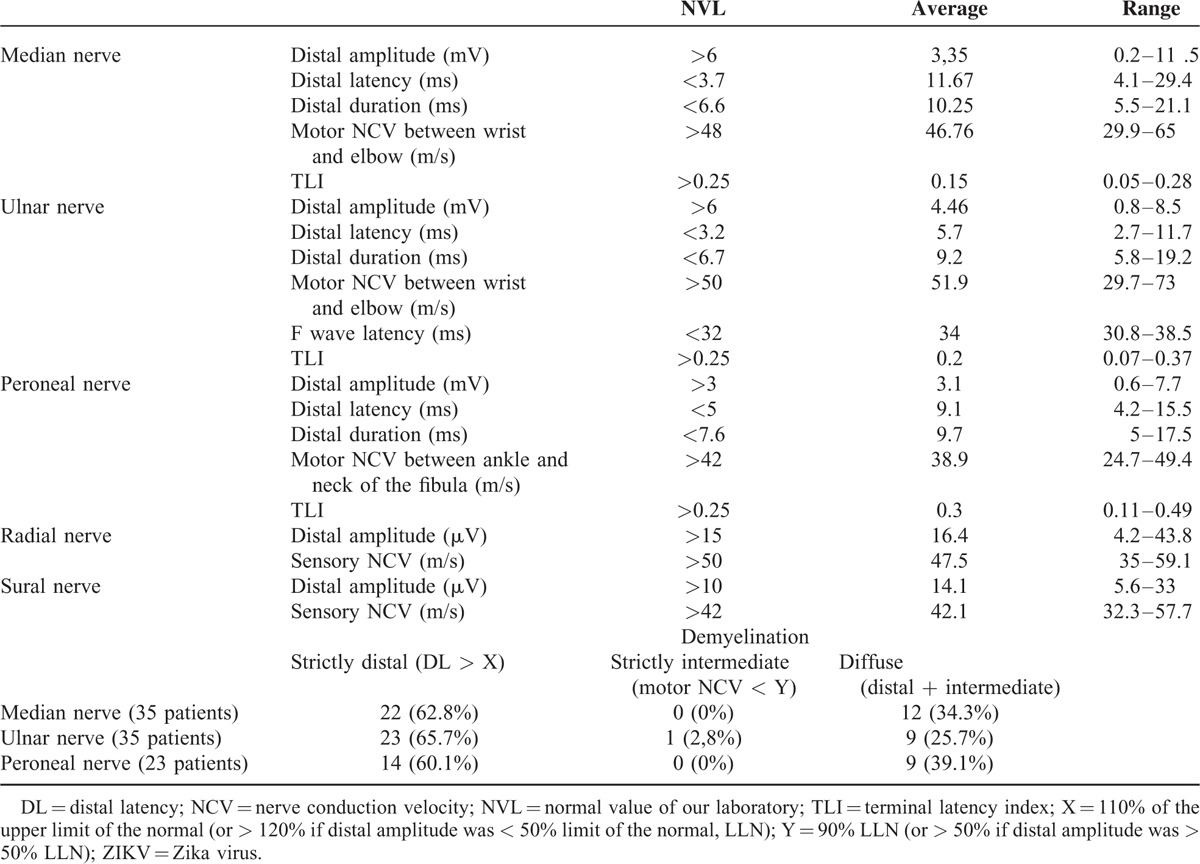
Electrophysiological Data (and Pattern of Demyelination) of Patients With GBS After ZIKV Infection

**FIGURE 2:**
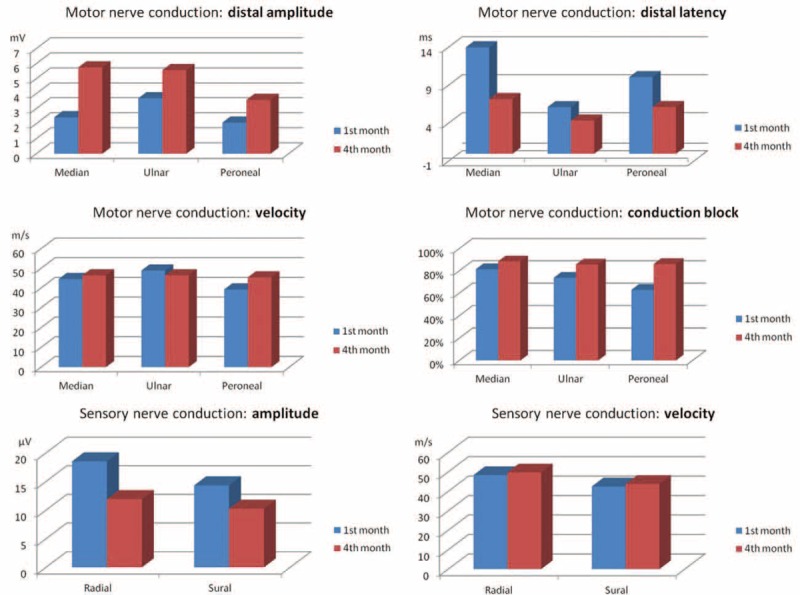
Improvement of ENMG for motor and sensitive nerves between the first and the fourth months (m/s = meter/second; ms = millisecond; mV = millivolt; μV = microvolt). ENMG = electroneuromyogram.

A second ENMG was performed during the 4th month in 22 of the patients. In comparison with the first one, results showed a clear improvement of the distal conduction abnormalities (*p* < 0.001), with reduction of the prolonged distal latencies and near-normalization of CMAP amplitudes (Figure 2).

### Biological Data

All patients underwent lumbar puncture on the day of their admission to hospital, or the day after (1 was done on the 8th day): 90% of the patients had a high protein level (normal value: 18–58 mg/dL), ranging from 31 to 1321 mg/dL (average: 228 mg/dL). Average white cell count was 5.5/μL (0 to 28). Culture for CSF samples was negative for microorganisms in all patients, and no patient had a low glucose level. We performed DENV RT-PCR on 9 CSF samples and ZIKV RT-PCR on 35 samples (all negative).

In blood samples, DENV PCR was done for 40 patients (all negative, except for 1 but with negative NS1 antigen); ZIKV PCR was done for 35 patients (negative) and DENV NS1 antigen for 24 patients (all negative except for 1). Among the blood serologies performed at admission, only 1 patient had positive IgG for *C jejuni* (the 1 born in France).

For the anti-ganglioside antibodies, only 7 sera were strictly positive: 2 for GM2, GD1a, and GD1b; 3 for GD1a, and 2 for GD1b; none were strictly positive for GM1 and GQ1b.

### Radiological Data

The 38 brain CT-scans carried out at baseline were normal. Five patients had a brain MRI: 2 presented a contrast enhancement of cranial nerves (1 with facial palsy; the other with facial diplegia and swallowing troubles). Eight patients had spine MRI: 3 presented contrast enhancement of nerve roots (1 with cranial nerve enhancement on brain MRI, but no motor weakness of the limbs; the other 2 patients were quadriparetic).

## DISCUSSION

We report here the first and largest series of Guillain–Barré syndrome (GBS) possibly related to ZIKV (42 cases). Classical GBS symptoms were preceded by symptoms of ZIKV infection (cutaneous eruption, arthralgia, fever, conjunctival hyperemia) during the month before the first neurological signs appeared. Motor disability could initially be severe, necessitating admission in intensive care unit and tracheotomy for 38% and 24% of the patients. The clinical phenotype matched what has been described for acute motor axonal neuropathy (AMAN). ENMG assessments performed during the first week of the disease showed marked distal motor nerve conduction alterations, which could help to explain the muscle weakness. Prolonged distal latencies and reduced distal CMAP amplitude may initially have been interpreted as demyelinating conduction slowing and block, leading to fallacious classification of the GBS pattern as acute inflammatory demyelinating neuropathy (AIDP) with axonal degeneration. However, the disappearance of the distal motor conduction alterations during follow-up (without development of abnormal temporal dispersion or conduction slowing in intermediate nerve segments) was consistent with what has been described as “reversible conduction failure” in AMAN.^28,29^ In these cases, the term “axonal” should be associated not with axonal degeneration but rather with transitory, promptly reversible, dysfunction of the excitable axolemma (presumably due to pathological changes at the nodes or paranodes).^30,31^ In our patients, such nodal/paranodal dysfunctions generally tended to be located in distal motor nerve endings.

Whereas ZIKV is highly neurotropic in mice,^32^ no neurological complications had been described in humans before 2014; the reasons why GBS was not described in previous cases of Zika fever before are not altogether clear. One evident possibility is the high number of patients affected during the French Polynesian outbreak, which was multiplied by 20 during this epidemic (the annual usual incidence of GBS being 1 to 2 patients in French Polynesia).^15^ Another noteworthy point involves the demographical data. First of all, the sex ratio is pronouncedly in favor of males (2.82), whereas it is 1.26 in general population.^17^ The mean age is 46, which is a little younger than in the general population.^17^ All of our cases of GBS (except 2) were observed in native Polynesians, even though the virus equally affected the many different ethnic groups living in French Polynesia. It is important to note that the patient from France was the only 1 to have a positive serology for *C jejuni*, which could consequently represent the “expected annual GBS case.” This observation may suggest a particular susceptibility of the Polynesian population to development of GBS after ZIKV infection. It is generally accepted that Polynesia was first settled by peoples from Southeast Asia.^33^ We also know that a number of medical differences exist between European and Austronesian populations: for example, Polynesians and Maoris are generally not affected by “European autoimmune diseases” but present specific autoimmune disorders.^34^ HLA (human leukocyte antigene), with highly polymorphic gene clusters that affect immune responses to infection and are implicated in autoimmune diseases, is known to be a candidate risk factor in GBS. In a recent meta-analysis, no significant associations were found between HLA-DQB1 polymorphisms and the risk of GBS in Asian and Caucasian populations; however, 2 associations approached significance: HLA-DQB1∗030× in Asian patients and HLA-DQB1∗060× in all patients.^35^ It is likely that the HLA data generated from the patients of our study will help to clarify the hypothesis of an interaction between the Polynesian HLA system and flaviviruses. ZIKV infection has recently spread to the Americas: 14,835 cases of exanthematous illness caused by ZIKV infection reported in Brazil (between 15 February and 25 June 2015), and many other cases have been described in several different countries in South and Central America. Recently (January 2016), the ECDC (European Centre for Disease Prevention and Control) reported unusual increases in cases of GBS occurring during ZIKV outbreak: 121 cases in Brazil, 46 cases in El Salvador, 15 cases in Venezuela, and a possible case in Caribbean (Martinique).^36^ If we have no details, these observations support a temporal and spatial association between the occurrence of GBS and ZIKV outbreak, comparable to that observed in French Polynesia; however, the number of cases observed in these densely countries is proportionally lower than those observed in French Polynesia (that is sparsely populated): this could suggest an increase chance for Polynesian people of developing GBS after ZIKV infection. Another particularity about ZIKV is the unusual increase of cases of congenital microcephaly (a severe neurological complication never before described during ZIKV infection) observed in Brazil (but not in French Polynesia), without any clear explanation for the moment.^36^

It is also possible that the high incidence of ZIKV-related GBS observed in Polynesian people (and now American populations) is due to some specificities of this arbovirus. A recent study has confirmed that ZIKV has gone through several recombination events.^37^ After emerging from Central Africa, it moved to West Africa, Asia and then Oceania (without a clear preference for host and vector species), and the Americas.^37^ It was recently shown that the French Polynesian and the American ZIKV belong to the Asian lineage.^38,39^ As for other infectious agents, in ZIKV-related GBS, an immune origin is suspected; there could exist molecular mimicry between ZIKV surface protein and gangliosides, and recent data on the recombination of this virus may explain the emergence of GBS (not described in the past). Moreover, many patients affected by GBS with recent ZIKV infection had previously developed an immunity against DENV:^40^ the risk of developing GBS could be subtended by a specific sequence of past DENV immunization and recent ZIKV infection. In our patients, we can suspect a DENV3 serotype for several reasons. First, when the ZIKV epidemic started, French Polynesia was experiencing an ongoing outbreak of DENV3. Second, there was no patient born after 1987 (the last previous period of circulation of DENV3 being 1989–1996),^14^ so all of those included in our study had potentially been exposed to DENV3. Third, this DENV3 epidemic spared the Marquesas archipelago (unpublished data), and no case of GBS has been observed in this location; as regards the man born in French Guyana, DENV3 is present in that French overseas department.^14^

To conclude, the ability of the virus to be transmitted by various species of *Aedes* mosquitoes is a problem for public health. More specifically, ZIKV is transmitted by *Aedes Aegypti*, but recently *Aedes albopictus* (also found in Mediterranean countries) was found to be another potential vector for the transmission of ZIKV ^41^; in French Polynesia, *Aedes polynesiensis* is also suspected.^13^ As a result, ZIKV epidemics can be expected in countries infested by these mosquitoes.

## CONCLUSION

We report the first and largest series of GBS probably related to ZIKV: this clustering of cases of GBS in French Polynesia is very uncommon (based on the usual experience in this country) and was observed in timing to coincide with a Zika virus outbreak. These observations may suggest potential existence of interactions between ZIKV and the Polynesian HLA syndrome and /or the consequence of several recombination events of this virus; however, the data collected in our retrospective (and descriptive) study are not sufficient to confirm this hypothesis. As a consequence, interactions between ZIKV, DENV, and Polynesian HLA system should be further investigated in view of more clearly imputing this epidemic to ZIKV infection and of better understanding the underlying physiopathology. Although the recently discovered temporal and spatial association between ZIKV outbreak and an unusually high number of GBS cases remains very suspicious, ZIKV has yet to be confirmed as the causative agent. However, since the recent spread of this virus to the Americas, the situation clearly calls for increased vigilance, particularly in countries where *Aedes* mosquitoes are present.
